# Association of Periprocedural GLP-1 Receptor Agonist Therapy With 1-Year Major Adverse Cardiovascular Events After Carotid Artery Stenting: A Propensity-Matched Analysis

**DOI:** 10.1016/j.jscai.2026.105387

**Published:** 2026-04-24

**Authors:** Abdullah Ghuman, Maumita Das

**Affiliations:** Department of Internal Medicine, TidalHealth Peninsula Regional, Salisbury, Maryland

**Keywords:** atherosclerotic cardiovascular disease, carotid artery stenting, glucagon-like peptide-1 receptor agonists, major adverse cardiovascular events, mortality, propensity score matching

## Abstract

**Background:**

Major adverse cardiovascular events (MACE) following carotid artery stenting (CAS) remain clinically significant. Pharmacological strategies to reduce postprocedural cardiovascular risk beyond antiplatelet therapy and statins are limited. Glucagon-like peptide-1 receptor agonists (GLP-1 RA) reduce MACE in patients with type 2 diabetes and established atherosclerotic cardiovascular disease; however, data evaluating GLP-1 RA therapy specifically in CAS populations are limited.

**Methods:**

Using the TriNetX Global Collaborative Network, we conducted a retrospective cohort study of adults undergoing CAS between 2015 and 2023. Periprocedural GLP-1 RA exposure was defined as at least 1 documented prescription within ±12 months of CAS. The primary outcome was 1-year composite MACE (myocardial infarction, cerebral infarction, or all-cause mortality). Propensity score matching (1:1 nearest-neighbor) balanced 41 baseline covariates, yielding 899 matched pairs.

**Results:**

Before matching, 906 patients received periprocedural GLP-1 RA therapy and 29,476 patients had no GLP-1 RA exposure. After matching, 899 pairs (1798 patients) were analyzed. Periprocedural GLP-1 RA therapy was associated with lower 1-year composite MACE (357/899 [39.7%] vs 401/899 [44.6%]; risk ratio, 0.89; 95% CI, 0.80-0.99; *P* = .04), representing an absolute risk reduction of 4.9% (number needed to treat = 20). The association with composite MACE appeared largely attributable to lower observed all-cause mortality (35/899 [3.9%] vs 80/899 [8.9%]; risk ratio, 0.44; 95% CI, 0.30-0.64; *P* < .001), whereas myocardial infarction and cerebral infarction did not differ significantly between groups.

**Conclusions:**

Periprocedural GLP-1 RA therapy was associated with lower 1-year MACE following CAS. These findings are hypothesis-generating and warrant prospective evaluation in carotid revascularization populations.

## Introduction

Carotid artery stenting (CAS) is an established revascularization strategy for patients with significant carotid stenosis, particularly those at elevated surgical risk. Despite advances in technique, embolic protection devices, and operator experience, periprocedural stroke or death rates with CAS remain higher than with carotid endarterectomy in symptomatic patients. In the CREST trial, periprocedural stroke or death occurred in 6.0% of symptomatic patients undergoing CAS versus 3.2% with endarterectomy (hazard ratio [HR], 1.89; 95% CI, 1.11-3.21; *P* = .02).^1^ In ICSS, rates were 7.4% vs 3.4% (risk ratio [RR], 2.16; 95% CI, 1.40-3.34; *P* < .001).^2^

Beyond periprocedural neurologic risk, patients undergoing carotid revascularization frequently have diffuse polyvascular disease and remain vulnerable to myocardial infarction, recurrent stroke, and death during follow-up. In a Vascular Quality Initiative analysis of 93,736 patients, polyvascular disease was present in 47%, and was independently associated with perioperative myocardial infarction or death (odds ratio, 1.59; 95% CI, 1.40-1.81) and reduced 5-year survival.^3^ Current pharmacological strategies focus primarily on antiplatelet therapy and statin optimization, yet adjunctive therapies targeting systemic cardiovascular risk reduction following CAS remain limited.

Glucagon-like peptide-1 receptor agonists (GLP-1 RA) have emerged as an important medication class for cardiovascular risk reduction beyond glycemic control. Large cardiovascular outcome trials, including LEADER, SUSTAIN-6, and REWIND, demonstrated that GLP-1 RA reduce major adverse cardiovascular events (MACE) in patients with type 2 diabetes and established atherosclerotic cardiovascular disease.^4^, ^5^, ^6^ A meta-analysis of 8 cardiovascular outcome trials encompassing 60,080 patients demonstrated a 14% reduction in MACE (HR, 0.86; 95% CI, 0.80-0.93), 16% reduction in fatal and nonfatal stroke (HR, 0.84; 95% CI, 0.76-0.94), and 12% reduction in all-cause mortality (HR, 0.88; 95% CI, 0.82-0.94).^7^ This stroke reduction is particularly relevant to the carotid population, as a separate meta-analysis of 28 trials (74,148 patients) confirmed a 17% reduction in adverse cerebrovascular outcomes (RR, 0.83; 95% CI, 0.76-0.91), with benefits most pronounced for longer-acting formulations.^8^ Proposed mechanisms include antiinflammatory effects, improved endothelial function, and plaque stabilization.^9^^,^^10^ Liraglutide, semaglutide, and dulaglutide have FDA approval to reduce MACE risk in adults with type 2 diabetes and established cardiovascular disease.^11^

Given the high burden of polyvascular atherosclerosis in patients undergoing CAS, and the unique periprocedural stroke risk related to embolic phenomena during plaque manipulation, GLP-1 RA may represent a biologically plausible adjunct for reducing postprocedural cardiovascular events. GLP-1 RA demonstrated effects on plaque stabilization, inflammation, and endothelial function that may be particularly relevant in this population.^9^^,^^10^ However, data evaluating GLP-1 RA therapy specifically in CAS populations are lacking. We, therefore, performed a propensity score–matched analysis using the TriNetX Global Collaborative Network to evaluate the association between periprocedural GLP-1 RA use and 1-year MACE following CAS.

## Materials and methods

### Study design and data source

We conducted a retrospective cohort study using propensity score matching with data from the TriNetX Global Collaborative Network, a federated database aggregating electronic health record data from 110 health care organizations comprising more than 100 million individuals, predominantly from the United States. TriNetX maps data to standardized terminologies, including International Classification of Diseases, Ninth and Tenth Revision, Clinical Modification (ICD-9-CM, ICD-10-CM), Current Procedural Terminology, Systematized Nomenclature of Medicine, and RxNorm codes. Mortality data are ascertained via contributing health systems and external death registries. The database complies with the Health Insurance Portability and Accountability Act as a limited dataset with deidentified patient data; institutional review board approval was not required. This study adhered to the Strengthening the Reporting of Observational Studies in Epidemiology (STROBE) guideline.^12^

### Study population

Adults aged ≥18 years who underwent CAS between January 1, 2015, and December 31, 2023, were identified using the Systematized Nomenclature of Medicine–CT code 233405004 (insertion of carotid artery stent), ICD-9-CM code 00.63 (percutaneous insertion of carotid artery stent[s]), and Current Procedural Terminology (CPT) codes 37215, 37216, 37217, 37218, and 1022228; codes are defined in Supplemental Table S1. The index date was defined as the date of the first CAS procedure.

### Exposure definition

Periprocedural GLP-1 RA exposure was defined as at least 1 documented prescription for a GLP-1 receptor agonist (semaglutide [RxNorm: 1991302], liraglutide [RxNorm: 475968], lixisenatide [RxNorm: 1440051]) or the dual GIP/GLP-1 receptor agonist tirzepatide (RxNorm: 2601723) within ±12 months of the index procedure. This window was selected to capture patients on established therapy prior to CAS and those initiated postprocedurally, reflecting real-world prescribing patterns. Medication adherence, treatment duration, and persistence beyond the initial prescription could not be verified from electronic health record data. The indication for GLP-1 RA prescription (type 2 diabetes mellitus [T2DM], obesity, or cardiovascular risk reduction) could not be determined from available data. The proportion of patients initiated on GLP-1 RA therapy before versus after the index procedure could not be determined within the TriNetX platform. Controls had no GLP-1 RA or tirzepatide prescription within the same window. Dulaglutide and exenatide were not included because of limitations in RxNorm code availability within the TriNetX query interface at the time of analysis; however, the included agents (semaglutide, liraglutide) have the most robust cardiovascular outcome trial evidence from LEADER and SUSTAIN-6.^4^^,^^5^

### Outcomes

The primary outcome was 1-year composite MACE, defined as myocardial infarction (ICD-10-CM: I21), cerebral infarction (ICD-10-CM: I63), or all-cause mortality occurring from day 1 through day 365 after CAS. The cerebral infarction outcome specifically captured ischemic stroke events. Cerebral infarction was identified by ICD-10-CM code I63, which typically requires imaging confirmation and clinical documentation, though adjudication of individual events was not possible. Periprocedural events occurring on day 0 were excluded to focus on postprocedural outcomes. Patients with prior myocardial infarction or cerebral infarction were included in the analysis, with prior events treated as baseline covariates; only incident events occurring after the index procedure were counted as outcomes. Because prior myocardial infarction and cerebral infarction were permitted at baseline, the composite reflects incident events in a population enriched for prior cerebrovascular disease. Secondary outcomes included the individual components of the composite analyzed separately.

### Propensity score matching

We performed 1:1 nearest-neighbor propensity score matching using a greedy algorithm without replacement. The propensity score was estimated via logistic regression with GLP-1 RA exposure as the dependent variable and 41 baseline covariates measured prior to or at the index procedure. Covariates included diagnoses (21 variables: obesity, T2DM, hyperlipidemia, hypertensive diseases, carotid artery stenosis laterality, cerebral infarction, history of transient ischemic attack, chronic kidney disease stages, and atrial fibrillation), medications (17 variables: antiplatelet agents, antihypertensives, statins, PCSK9 inhibitors, and ezetimibe), and laboratory values (3 variables: body mass index [BMI], estimated glomerular filtration rate, and hemoglobin A1c [HbA1c]). The complete list of all 41 covariates with standardized mean differences (SMD) before and after matching is provided in Supplemental Table S2. The TriNetX platform applies greedy nearest-neighbor matching with a caliper of 0.1 pooled standard deviations of the propensity score. Covariate balance was assessed using SMD, with SMD ≤0.10 indicating adequate balance. Seven patients in the GLP-1 RA cohort could not be matched and were excluded.

### Statistical analysis

Baseline characteristics were summarized as means with standard deviations for continuous variables and frequencies with percentages for categorical variables. Patients with missing data for matching covariates were excluded from the propensity score model. RR with 95% CI were calculated based on cumulative 1-year event incidence as provided by the TriNetX Measures of Association analysis. Absolute risk reduction (ARR) and number needed to treat (NNT) (NNT = 1/ARR) were derived from observed event rates. All analyses were performed within the TriNetX platform using built-in statistical software; patient-level data were not extracted. Two-sided *P* <.05 was considered statistically significant.

### Sensitivity analysis for unmeasured confounding

To assess the robustness of observed associations to potential unmeasured confounding, we calculated E-values as described by VanderWeele and Ding.^12^ The E-value represents the minimum strength of association that an unmeasured confounder would need to have with both the exposure and outcome, conditional on measured covariates, to fully explain the observed association. E-values were calculated for point estimates and for the CI limit closest to the null.

## Results

### Study population and propensity score matching

**Figure 1:**
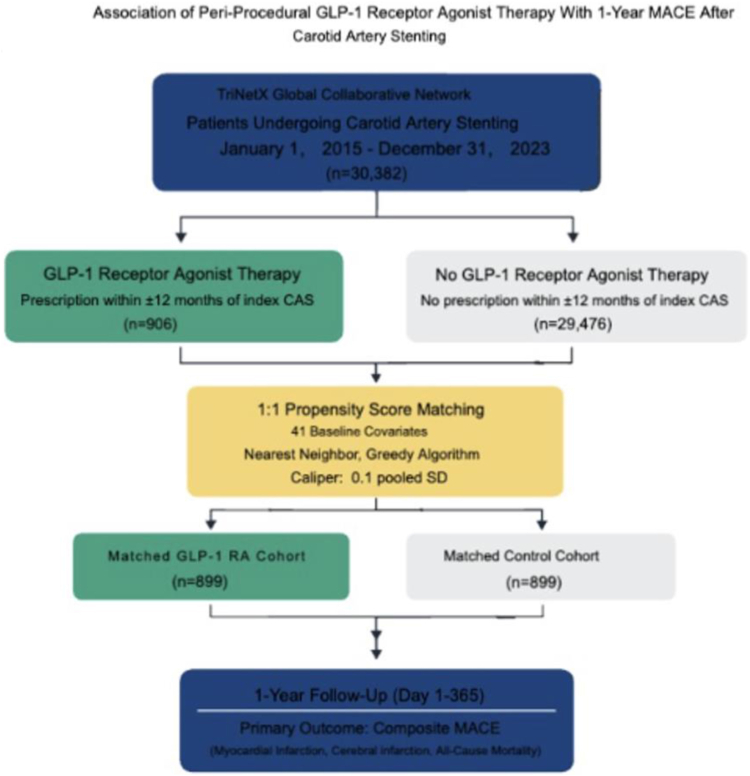
**Study flow diagram.** Flow diagram illustrating patient selection from the TriNetX Global Collaborative Network. Patients undergoing carotid artery stenting (CAS) between January 1, 2015, and December 31, 2023, were identified and stratified by periprocedural glucagon-like peptide-1 (GLP-1) receptor agonist (RA) exposure (prescription within ±12 months of index procedure). After 1:1 propensity score matching using 41 baseline covariates with a caliper of 0.1 pooled standard deviations, 899 matched pairs were analyzed for the primary outcome of 1-year composite major adverse cardiovascular events (MACE).

**Figure 2:**
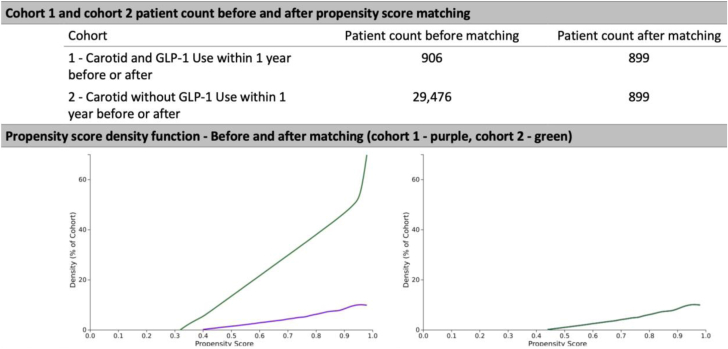
**Propensity score distribution before and after matching.** Propensity score density plots demonstrating the distribution of propensity scores for patients with periprocedural glucagon-like peptide-1 (GLP-1) receptor agonist (RA) exposure (purple) and controls without GLP-1 RA exposure (green) before matching (left panel) and after 1:1 nearest-neighbor propensity score matching (right panel). Before matching, substantial differences in propensity score distributions were observed between the GLP-1 RA group (n = 906) and the control group (n = 29,476), reflecting baseline imbalances in covariates. After matching, 899 pairs (1798 patients) demonstrated improved overlap in propensity score distributions, indicating adequate covariate balance. Propensity scores were estimated using logistic regression with 41 baseline covariates, and matching was performed using a greedy algorithm with a caliper of 0.1 pooled standard deviations.

From the TriNetX Global Collaborative Network, we identified 30,382 patients who underwent CAS. Of these, 906 patients received periprocedural GLP-1 RA therapy within ±12 months of the index procedure, and 29,476 patients had no GLP-1 RA exposure within the periprocedural window. After 1:1 nearest-neighbor propensity score matching, 899 matched pairs (1798 patients) were available for analysis; propensity score distributions demonstrated improved overlap between groups following matching (Figures 1 and 2). Seven patients in the GLP-1 RA cohort could not be matched. All baseline covariates achieved adequate balance after matching (SMD ≤0.10) except for BMI (SMD = 0.37) and HbA1c (SMD = 0.31), which remained imbalanced with higher values in the GLP-1 RA group (Table 1).

**Table 1:** Baseline characteristics of patients undergoing carotid artery stenting before and after propensity score matching

Characteristic	Before matching			After matching		
	GLP-1 (n = 906)	No GLP-1 (n = 29,476)	SMD	GLP-1 (n = 899)	No GLP-1 (n = 899)	SMD
Diagnoses						
Obesity (E66.9)	490 (54.1%)	4729 (16.2%)	0.866	483 (53.7%)	478 (53.2%)	0.011
Type 2 diabetes (E11)	792 (87.4%)	9435 (32.2%)	1.361	785 (87.3%)	797 (88.7%)	0.041
Pure hypercholesterolemia (E78.0)	361 (39.8%)	6673 (22.8%)	0.374	355 (39.5%)	344 (38.3%)	0.025
Mixed hyperlipidemia (E78.2)	436 (48.1%)	5664 (19.4%)	0.639	429 (47.7%)	425 (47.3%)	0.009
Hypertensive diseases (I10-I1A)	854 (94.3%)	21610 (73.9%)	0.580	847 (94.2%)	855 (95.1%)	0.040
Carotid artery stenosis (I65.2)	832 (91.8%)	22433 (76.7%)	0.425	825 (91.8%)	832 (92.5%)	0.029
Cerebral infarction (I63)	437 (48.2%)	11539 (39.4%)	0.178	435 (48.4%)	441 (49.1%)	0.013
History of TIA/stroke (Z86.73)	290 (32.0%)	5777 (19.7%)	0.283	288 (32.0%)	297 (33.0%)	0.021
Chronic kidney disease (N18)	323 (35.7%)	5712 (19.5%)	0.367	320 (35.6%)	311 (34.6%)	0.021
Atrial fibrillation/flutter (I48)	197 (21.7%)	4711 (16.1%)	0.144	194 (21.6%)	199 (22.1%)	0.013
Medications						
Aspirin	820 (90.5%)	20764 (71.0%)	0.511	813 (90.4%)	815 (90.7%)	0.008
Clopidogrel	813 (89.7%)	20134 (68.8%)	0.534	806 (89.7%)	825 (91.8%)	0.073
Atorvastatin	714 (78.8%)	15933 (54.5%)	0.534	707 (78.6%)	723 (80.4%)	0.044
Rosuvastatin	329 (36.3%)	4701 (16.1%)	0.473	323 (35.9%)	294 (32.7%)	0.068
Ezetimibe	192 (21.2%)	2209 (7.6%)	0.396	187 (20.8%)	182 (20.2%)	0.014
Lisinopril	458 (50.6%)	8440 (28.8%)	0.455	453 (50.4%)	447 (49.7%)	0.013
Losartan	327 (36.1%)	5038 (17.2%)	0.437	323 (35.9%)	335 (37.3%)	0.028
Laboratory values						
BMI, kg/m^2^	32.5 ± 6.5	28.2 ± 5.9	0.700	32.5 ± 6.5	30.2 ± 6.1	0.366
eGFR, mL/min/1.73 m^2^	69.2 ± 27.2	72.2 ± 27.3	0.112	69.1 ± 27.0	68.7 ± 27.2	0.012
HbA1c, %	7.5 ± 1.8	6.4 ± 1.4	0.720	7.5 ± 1.8	7.0 ± 1.6	0.309

Values are presented as n (%) for categorical variables and mean ± SD for continuous variables.

Propensity score matching was performed using 1:1 nearest-neighbor matching with a greedy algorithm and a caliper of 0.1 pooled standard deviations. Covariates were measured prior to or at the index procedure date.

SMD ≤0.10 indicates adequate balance between groups. After matching, BMI and HbA1c remained imbalanced (SMD >0.10), with the GLP-1 receptor agonist group having higher values for both parameters.

BMI, body mass index; eGFR, estimated glomerular filtration rate; GLP-1, glucagon-like peptide-1; HbA1c, hemoglobin A1c; SMD, standardized mean difference; TIA, transient ischemic attack.

### Baseline characteristics

Baseline characteristics of the propensity-matched cohort are presented in Table 1. The matched cohort demonstrated high cardiovascular risk burden, with T2DM present in 87.3% of the GLP-1 RA group and 88.7% of controls. Prior cerebral infarction was documented in 48.4% and 49.1%, respectively. Rates of hypertensive diseases (94.2% vs 95.1%), mixed hyperlipidemia (47.7% vs 47.3%), chronic kidney disease (35.6% vs 34.6%), and atrial fibrillation (21.6% vs 22.1%) were similarly distributed between groups. Concomitant cardiovascular medications were well balanced, including aspirin (90.4% vs 90.7%), clopidogrel (89.7% vs 91.8%), and atorvastatin (78.6% vs 80.4%). Despite higher BMI (32.5 vs 30.2 kg/m^2^; SMD = 0.37) and HbA1c (7.5% vs 7.0%; SMD = .31) values following matching, the GLP-1 RA group demonstrated lower observed event rates, suggesting that residual imbalance in these metabolic parameters does not fully account for the association.

### Primary outcome

At 1 year, the primary composite outcome of MACE occurred in 357 of 899 patients (39.7%) in the GLP-1 RA group compared with 401 of 899 patients (44.6%) in the control group (RR, 0.89; 95% CI, 0.80-0.99; *P* = .04) (Table 2, Central Illustration). This corresponded to an ARR of 4.9% (95% CI, 0.3%-9.5%) and an NNT of 20 to prevent 1 MACE event at 1 year.

**Table 2:** Clinical outcomes at 1 year after carotid artery stenting in propensity-matched cohorts

Outcome	GLP-1 RA (n = 899)	Control (n = 899)	Risk ratio (95% CI)	*P* value	ARR	NNT
Primary outcome						
Composite MACE	357 (39.7)	401 (44.6)	0.89 (0.80-0.99)	.04	4.9%	20
Secondary outcomes						
All-cause mortality	35 (3.9)	80 (8.9)	0.44 (0.30-0.64)	.001	5.0%	20
Cerebral infarction	291 (32.4)	317 (35.3)	0.92 (0.81-1.05)	.20	2.9%	–
Myocardial infarction	77 (8.6)	83 (9.2)	0.93 (0.69-1.25)	.62	0.6%	–

Values are presented as n (%) unless otherwise indicated. Outcomes were assessed from day 1 through day 365 after the index procedure; periprocedural events occurring on day 0 were excluded.

The primary outcome was composite MACE, defined as the first occurrence of myocardial infarction, cerebral infarction, or all-cause mortality. Individual components were analyzed as exploratory secondary outcomes.

Risk ratios with 95% CI were calculated comparing the GLP-1 RA group with the control group (reference). *P* values <.05 were considered statistically significant.

ARR, absolute risk reduction; GLP-1 RA, glucagon-like peptide-1 receptor agonist; MACE, major adverse cardiovascular event; NNT, number needed to treat.

**Central Illustration:**
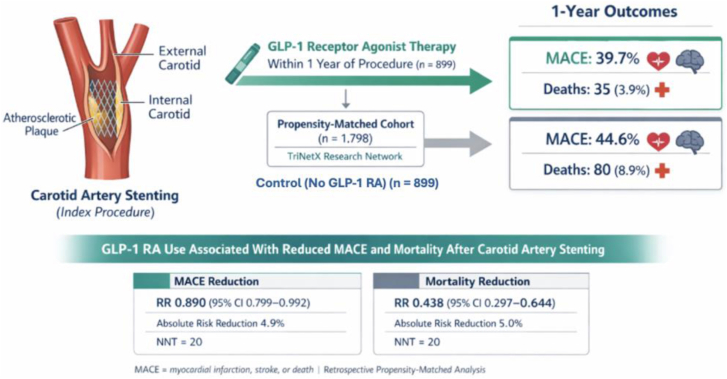
**Association of periproceduralGLP-1 therapy with reduced MACE and mortality after carotid artery stenting.** GLP-1 RA, glucagon-like peptide-1 receptor agonist; MACE, major adverse cardiovascular events; NNT, number needed to treat; RR, risk ratio.

### Secondary outcomes

Individual components of the composite MACE end point were analyzed as secondary outcomes (Table 2). The association with composite MACE appeared largely attributable to lower observed all-cause mortality: 35 patients (3.9%) in the GLP-1 RA group vs 80 patients (8.9%) in the control group (RR, 0.44; 95% CI, 0.30-0.64; *P* < .001), representing an ARR of 5.0%. Cerebral infarction occurred in 291 patients (32.4%) versus 317 patients (35.3%) (RR, 0.92; 95% CI, 0.81-1.05; *P* = .20). Myocardial infarction occurred in 77 patients (8.6%) versus 83 patients (9.2%) (RR, 0.93; 95% CI, 0.69-1.25; *P* = .62). Differences in myocardial infarction and cerebral infarction were not statistically significant.

### Sensitivity analysis

E-value analysis demonstrated variable robustness to unmeasured confounding across outcomes. For the primary composite MACE end point (RR, 0.89), the E-value was 1.38 (E-value for CI closest to null: 1.06), indicating that relatively modest unmeasured confounding could explain this association, warranting cautious interpretation. For all-cause mortality (RR, 0.44), the E-value was 3.90 (E-value for CI closest to null: 2.48) (Table 3).^12^ Although the mortality association demonstrated a higher E-value, unmeasured confounding cannot be excluded, particularly given the observational design and potential for selection bias.

**Table 3:** Sensitivity analysis for unmeasured confounding using E-values

Analysis	Outcome	Effect estimate	E-value (point)	E-value (CI)	Interpretation
Primary	Composite MACE	RR 0.89	1.38	1.06	Modest unmeasured confounding could explain
Primary	All-cause mortality	RR 0.44	3.90	2.48	Strong confounding required to explain
Primary	Cerebral infarction	RR 0.92	1.32	–	Not significant; E-value less relevant
Primary	Myocardial infarction	RR 0.93	1.27	–	Not significant; E-value less relevant

E-values were calculated as described by VanderWeele and Ding^12^ to assess the robustness of observed associations to potential unmeasured confounding. The E-value ​represents ​the minimum strength of association, on the RR scale, that an unmeasured confounder would need to have with both the exposure (GLP-1 RA therapy) and the outcome to fully explain away the observed association, conditional on measured covariates. The E-value for the CI ​represents ​the minimum confounding strength needed to shift the CI to include the null. Higher E-values ​indicate ​greater robustness to unmeasured confounding. As a benchmark, E-values exceeding 2.0 are ​generally considered ​moderately robust, whereas values exceeding 3.0 suggest substantial robustness. E-values were not calculated for the CI of nonsignificant outcomes, as these associations could already be explained by chance alone.

E-value, evidence value for unmeasured confounding; MACE, major adverse cardiovascular events (myocardial infarction, cerebral infarction, or all-cause mortality); RR, risk ratio.

## Discussion

In this propensity score–matched analysis of patients undergoing CAS, periprocedural GLP-1 RA therapy was associated with lower 1-year composite MACE compared with matched controls. The association appeared largely attributable to lower observed all-cause mortality, whereas individual ischemic end points were not statistically different. To our knowledge, data evaluating GLP-1 RA therapy specifically in CAS populations are limited, and this analysis provides initial observational evidence in this high-risk population.

The CAS population has several distinguishing features that warrant specific investigation. Patients undergoing carotid revascularization have exceptionally high polyvascular disease burden (47% in the Vascular Quality Initiative registry), conferring elevated risk for both neurologic and systemic cardiovascular events.^3^ Unlike coronary or peripheral interventions, CAS carries a unique periprocedural stroke risk (6.0%-7.4% in symptomatic patients) related to embolic phenomena during manipulation of atherosclerotic plaque.^1^^,^^2^ GLP-1 RA have demonstrated preferential cerebrovascular benefits in meta-analyses, with 14% to 17% reductions in stroke.^7^^,^^8^ These stroke-protective effects may be particularly relevant in carotid atherosclerosis, where plaque vulnerability directly determines embolic risk during and after intervention. Despite these biological rationales, no prior studies have specifically evaluated GLP-1 RA therapy in carotid revascularization populations. Our study provides the first observational evidence that periprocedural GLP-1 RA therapy may be associated with improved outcomes following CAS, warranting prospective evaluation in this high-risk population.

These findings extend the established cardiovascular benefits of GLP-1 RA to a population undergoing carotid intervention. A recent meta-analysis of 21 randomized controlled trials encompassing 99,599 patients demonstrated that GLP-1 RA reduce all-cause mortality by 12%, cardiovascular mortality by 13%, and MACE by 14% compared with controls.^13^ The SELECT trial demonstrated that semaglutide reduces MACE by 20% in patients with established cardiovascular disease and overweight or obesity without diabetes (HR, 0.80; 95% CI, 0.72-0.90), with benefits observed irrespective of baseline glycemic status.^14^ The magnitude of the mortality association observed in our study exceeds that reported in randomized trials and should be interpreted cautiously, as it may reflect residual confounding or bias introduced by the exposure window.

The predominant association with mortality rather than ischemic end points warrants consideration. Our study may have been underpowered to detect differences in individual ischemic components, particularly given the modest effect sizes observed (RR, 0.92-0.93). Additionally, the 1-year follow-up period may be insufficient to observe the full antiatherosclerotic effects of GLP-1 RA therapy; in major cardiovascular outcome trials, Kaplan-Meier curves for MACE typically began separating at 12 to 18 months.^4^, ^5^, ^6^ The mortality association may also reflect mechanisms distinct from atherothrombotic protection. Meta-analyses suggest that GLP-1 RA may reduce heart failure events and other systemic complications, supporting potential mechanisms beyond atherothrombosis.^15^^,^^16^

The high baseline cardiovascular risk burden of our matched cohort (Table 1)—with approximately 87% to 89% having type 2 diabetes, 48% to 49% with prior cerebral infarction, and over 94% with hypertensive diseases—reflects the polyvascular nature of atherosclerotic disease in patients requiring carotid revascularization. However, the diabetes prevalence substantially exceeds that reported in carotid revascularization populations (28%-41%),^17^ likely reflecting selection bias inherent to the TriNetX platform and enrichment for diabetic patients given the periprocedural exposure definition. This limits generalizability to broader CAS populations. The observed 1-year MACE rates of approximately 40% to 45% are notably higher than those reported in randomized trials (9%-13%),^1^^,^^2^ reflecting our broader composite end point definition, the high prevalence of diabetes and prior cerebrovascular events, and the real-world nature of electronic health record data. Additionally, electronic health record coding practices may capture rule-out diagnoses or suspected events that would be adjudicated as nonevents in prospective trials. The higher-than-expected MACE rate may also reflect a predominantly symptomatic population or those with high-risk plaque features, which the TriNetX platform cannot adjudicate. Whether GLP-1 RA benefits differ between symptomatic versus asymptomatic patients, or those with versus without prior cerebrovascular events, remains unknown and warrants investigation in future prospective studies with adequate power for subgroup analyses.

### Limitations

Several limitations merit consideration. First, the observational design precludes causal inference. Although propensity score matching balanced 41 measured covariates, residual confounding from unmeasured variables cannot be excluded. BMI and HbA1c remained imbalanced after matching, though these differences would bias against the GLP-1 RA group.

Second, the periprocedural exposure window (±12 months) introduces potential immortal time bias. The bidirectional exposure window may introduce immortal time bias, as patients who died shortly after CAS would be less likely to receive postprocedural GLP-1 RA prescriptions. This may partially explain the magnitude of the observed mortality association. Although the bidirectional window captures patients prescribed GLP-1 RA before CAS, the proportion of preprocedural versus postprocedural initiators could not be determined. Future studies should employ time-dependent Cox regression analysis to address this bias.

Third, electronic health record data have inherent limitations. The federated network architecture aggregates data from hospital-based records rather than population-based registries, introducing potential selection bias. The fixed analytic interface does not support time-varying exposure methods, landmark analyses, or competing risks models. Clinical detail is limited; we could not ascertain symptomatic versus asymptomatic status, lesion characteristics, procedural details, or medication adherence. We could not assess whether treatment effects differed by symptomatic status, prior stroke or transient ischemic attack, degree of carotid stenosis, or plaque morphology—all of which may modify GLP-1 RA treatment effects. Future studies using individual patient-level data should evaluate effect modification by baseline stroke risk and symptomatic versus asymptomatic presentation.

Fourth, we could not distinguish between patients on established GLP-1 RA therapy prior to CAS versus those initiated after the procedure. Fifth, the inclusion of tirzepatide alongside pure GLP-1 RA may introduce heterogeneity, and the exclusion of dulaglutide and exenatide may affect generalizability.

Sixth, medication adherence and persistence could not be verified from electronic health record data. We could not determine whether GLP-1 RA were prescribed for type 2 diabetes, obesity, or cardiovascular risk reduction, though the high prevalence of diabetes in our cohort (87%-89%) suggests most prescriptions were for glycemic management. Additionally, we could not ascertain the proportion of patients who filled prescriptions, took medications as prescribed, or continued therapy throughout the follow-up period. These limitations may attenuate observed treatment effects toward the null if a substantial proportion of patients with documented prescriptions did not actually take the medication.

Seventh, cerebral infarction events were identified using ICD-10-CM code I63, which requires clinical documentation and typically imaging confirmation. However, patients with undiagnosed or subclinical strokes not captured in billing codes would be missed, potentially underestimating the true event rate. Additionally, we could not adjudicate stroke severity, mechanism (embolic vs thrombotic), or distinguish between in-territory versus out-of-territory events relative to the treated carotid artery. This limitation applies equally to both groups and would be expected to bias results toward the null.

## Conclusion

Our findings may support consideration of GLP-1 RA therapy as part of comprehensive secondary prevention following carotid revascularization, particularly in patients with concomitant type 2 diabetes or obesity. Semaglutide has FDA-approved indications to reduce MACE risk in adults with established cardiovascular disease and obesity or overweight, and the SELECT trial demonstrated cardiovascular benefits independent of diabetes status.^14^ In this propensity score–matched analysis, periprocedural GLP-1 RA therapy was associated with lower 1-year MACE following CAS, though this association showed limited robustness to unmeasured confounding (E-value 1.38). The association appeared largely attributable to lower observed mortality, whereas individual ischemic end points did not differ significantly. These hypothesis-generating findings require confirmation in prospective studies designed to address the limitations of observational data.

## CRediT authorship contribution statement

**Abdullah Ghuman:** Writing – review & editing, Writing – original draft, Supervision, Project administration, Methodology, Investigation, Formal analysis, Data curation, Conceptualization. **Maumita Das:** Writing – review & editing, Writing – original draft, Validation, Software, Methodology, Investigation, Formal analysis, Data curation, Conceptualization.

## Footnotes

To access the supplementary material accompanying this article, visit the online version of the *Journal of the Society for Cardiovascular Angiography & Interventions* at 10.1016/j.jscai.2026.105387.
