# Supplementary material for: Association of Periprocedural GLP-1 Receptor Agonist Therapy With 1-Year Major Adverse Cardiovascular Events After Carotid Artery Stenting: A Propensity-Matched Analysis
Source: J Soc Cardiovasc Angiogr Interv. 2026 Apr 24;5(6):105387. doi: 10.1016/j.jscai.2026.105387 (PMC13404038; doi:10.1016/j.jscai.2026.105387)
Supplement: Supplementary Data [file mmc1.docx]

# Complete Code Definitions for Study Cohorts, Exposures, and Outcomes

Supplemental Table S1. Complete Code Definitions for Study Population, Exposure, and Outcomes

A. Inclusion Criteria: Carotid Artery Stenting Procedure Codes

| Code System | Code | Description |
| --- | --- | --- |
| SNOMED-CT | 233405004 | Insertion of carotid artery stent |
| ICD-9-CM | 00.63 | Percutaneous insertion of carotid artery stent(s) |
| CPT | 37215 | Transcatheter placement of intravascular stent(s), cervical carotid artery, open or percutaneous, including angioplasty, when performed, and radiological supervision and interpretation; with distal embolic protection |
| CPT | 37216 | Transcatheter placement of intravascular stent(s), cervical carotid artery, open or percutaneous, including angioplasty, when performed, and radiological supervision and interpretation; without distal embolic protection |
| CPT | 37217 | Transcatheter placement of intravascular stent(s), intrathoracic common carotid artery or innominate artery by retrograde treatment, open ipsilateral cervical carotid artery exposure, including angioplasty, when performed, and radiological supervision and interpretation |
| CPT | 37218 | Transcatheter placement of intravascular stent(s), intrathoracic common carotid artery or innominate artery, open or percutaneous antegrade approach, including angioplasty, when performed, and radiological supervision and interpretation |
| CPT | 1022228 | Transcatheter placement of intravascular stent(s), cervical carotid artery, open or percutaneous, including angioplasty, when performed, and radiological supervision and interpretation |

B. Exposure Definition: GLP-1 Receptor Agonist and GIP/GLP-1 Receptor Agonist Medications

Periprocedural exposure was defined as at least one documented prescription within ±12 months of the index CAS procedure.

| Code System | RxNorm Code | Medication | Drug Class |
| --- | --- | --- | --- |
| RxNorm | 1991302 | Semaglutide | GLP-1 receptor agonist |
| RxNorm | 475968 | Liraglutide | GLP-1 receptor agonist |
| RxNorm | 1440051 | Lixisenatide | GLP-1 receptor agonist |
| RxNorm | 2601723 | Tirzepatide | Dual GIP/GLP-1 receptor agonist |

C. Cohort Definitions

| Cohort | Definition | Patients Before Matching | Patients After Matching |
| --- | --- | --- | --- |
| Cohort 1 (Exposure) | CAS procedure AND ≥1 GLP-1 RA/tirzepatide prescription within ±12 months of index procedure | 906 | 899 |
| Cohort 2 (Control) | CAS procedure AND NO GLP-1 RA/tirzepatide prescription within ±12 months of index procedure | 29,476 | 899 |

D. Outcome Definitions

Outcomes were assessed from day 1 through day 365 after the index procedure. Periprocedural events occurring on day 0 were excluded.

| Outcome | Code System | Code | Description |
| --- | --- | --- | --- |
| <strong>Primary Composite (MACE)</strong> |  |  |  |
| Myocardial Infarction | ICD-10-CM | I21 | Acute myocardial infarction |
| Cerebral Infarction | ICD-10-CM | I63 | Cerebral infarction |
| All-Cause Mortality | Demographics | Deceased | Deceased status flag |
| <strong>Secondary Outcomes</strong> |  |  |  |
| Myocardial Infarction | ICD-10-CM | I21 | Acute myocardial infarction |
| Cerebral Infarction | ICD-10-CM | I63 | Cerebral infarction |
| All-Cause Mortality | Demographics | Deceased | Deceased status flag |

E. Propensity Score Matching Covariates

Diagnoses (21 covariates)

| Category | ICD-10-CM Code | Description |
| --- | --- | --- |
| Obesity | E66.9 | Obesity, unspecified |
| Diabetes | E11 | Type 2 diabetes mellitus |
| Hyperlipidemia | E78.0 | Pure hypercholesterolemia |
| Hyperlipidemia | E78.2 | Mixed hyperlipidemia |
| Hypertension | I10-I1A | Hypertensive diseases |
| Carotid Stenosis | I65.2 | Occlusion and stenosis of carotid artery |
| Carotid Stenosis | I65.21 | Occlusion and stenosis of right carotid artery |
| Carotid Stenosis | I65.22 | Occlusion and stenosis of left carotid artery |
| Carotid Stenosis | I65.23 | Occlusion and stenosis of bilateral carotid arteries |
| Carotid Stenosis | I65.29 | Occlusion and stenosis of unspecified carotid artery |
| Cerebrovascular Disease | I63 | Cerebral infarction |
| Cerebrovascular Disease | Z86.73 | Personal history of transient ischemic attack (TIA), and cerebral infarction without residual deficits |
| Chronic Kidney Disease | N18 | Chronic kidney disease (CKD) |
| Chronic Kidney Disease | N18.1 | Chronic kidney disease, stage 1 |
| Chronic Kidney Disease | N18.2 | Chronic kidney disease, stage 2 (mild) |
| Chronic Kidney Disease | N18.3 | Chronic kidney disease, stage 3 (moderate) |
| Chronic Kidney Disease | N18.4 | Chronic kidney disease, stage 4 (severe) |
| Chronic Kidney Disease | N18.5 | Chronic kidney disease, stage 5 |
| Chronic Kidney Disease | N18.6 | End stage renal disease |
| Chronic Kidney Disease | N18.9 | Chronic kidney disease, unspecified |
| Arrhythmia | I48 | Atrial fibrillation and flutter |

Medications (17 covariates)

| Category | RxNorm Code | Medication |
| --- | --- | --- |
| Antiplatelet | 1191 | Aspirin |
| Antiplatelet | 32968 | Clopidogrel |
| Antiplatelet | 1116632 | Ticagrelor |
| Antiplatelet | 613391 | Prasugrel |
| ACE Inhibitor | 29046 | Lisinopril |
| ARB | 52175 | Losartan |
| Statin | 83367 | Atorvastatin |
| Statin | 301542 | Rosuvastatin |
| Statin | 36567 | Simvastatin |
| Statin | 42463 | Pravastatin |
| Statin | 41127 | Fluvastatin |
| Statin | 6472 | Lovastatin |
| Statin | 861634 | Pitavastatin |
| PCSK9 Inhibitor | 1659152 | Alirocumab |
| PCSK9 Inhibitor | 1665684 | Evolocumab |
| PCSK9 Inhibitor | 2478335 | Evinacumab |
| Cholesterol Absorption Inhibitor | 341248 | Ezetimibe |

Laboratory Values (3 covariates)

| LOINC Code | Laboratory Test | Units |
| --- | --- | --- |
| 9083 | Body Mass Index (BMI) | kg/m² |
| 8001 | Glomerular filtration rate/1.73 sq M predicted (eGFR) by Creatinine-based formula (MDRD) | mL/min/1.73 m² |
| 9037 | Hemoglobin A1c/Hemoglobin total in Blood | % |

F. Index Event and Time Window

- Index Event: Date of first CAS procedure

- Exposure Window: ±12 months from index event (GLP-1 RA prescription)

- Outcome Window: Day 1 through Day 365 after index event

- Study Period: January 1, 2015 – December 31, 2023

G. Exclusion Criteria

- Patients whose index event occurred more than 20 years prior to analysis (0 patients excluded in both cohorts)

- Patients in the GLP-1 RA cohort who could not be matched (7 patients excluded)

Notes:

- All codes were mapped via the TriNetX platform using standardized terminologies (UMLS, NLM:RXNORM).

- The TriNetX Global Collaborative Network aggregates electronic health record data from 110 healthcare organizations.

- Propensity score matching used 1:1 nearest-neighbor matching with a greedy algorithm and caliper of 0.1 pooled standard deviations.

G. Statistical software and version used

The study was conducted using the TriNetX Research Network platform.

- Statistical Software: TriNetX Explore.
- Version: 3.0.
- Analysis Date: The report and analyses were generated on January 26, 2026.

This software provides a global, federated health research network that allows for real-world, real-time data analysis across 113 healthcare organizations. The platform's built-in "Compare Outcomes Analysis" tool was used to run the measures of association, survival analyses, and propensity score matching.

# Extended Covariate Balance Table - All 41 Variables

Supplemental Table S2. Baseline Characteristics of Patients Undergoing Carotid Artery Stenting Before and After Propensity Score Matching: Complete 41-Covariate Analysis

| Characteristic | Before Matching GLP-1 RA (n=906) | Before Matching No GLP-1 RA (n=29,476) | Before Matching SMD | After Matching GLP-1 RA (n=899) | After Matching No GLP-1 RA (n=899) | After Matching SMD |
| --- | --- | --- | --- | --- | --- | --- |
| <strong>Diagnoses</strong> |  |  |  |  |  |  |
| Obesity, unspecified (E66.9) | 490 (54.1%) | 4,729 (16.2%) | 0.866 | 483 (53.7%) | 478 (53.2%) | 0.011 |
| Type 2 diabetes mellitus (E11) | 792 (87.4%) | 9,435 (32.2%) | 1.361 | 785 (87.3%) | 797 (88.7%) | 0.041 |
| Pure hypercholesterolemia (E78.0) | 361 (39.8%) | 6,673 (22.8%) | 0.374 | 355 (39.5%) | 344 (38.3%) | 0.025 |
| Mixed hyperlipidemia (E78.2) | 436 (48.1%) | 5,664 (19.4%) | 0.639 | 429 (47.7%) | 425 (47.3%) | 0.009 |
| Hypertensive diseases (I10-I1A) | 854 (94.3%) | 21,610 (73.9%) | 0.580 | 847 (94.2%) | 855 (95.1%) | 0.040 |
| Occlusion and stenosis of carotid artery (I65.2) | 832 (91.8%) | 22,433 (76.7%) | 0.425 | 825 (91.8%) | 832 (92.5%) | 0.029 |
| Occlusion and stenosis of right carotid artery (I65.21) | 491 (54.2%) | 9,525 (32.6%) | 0.447 | 488 (54.3%) | 476 (52.9%) | 0.027 |
| Occlusion and stenosis of left carotid artery (I65.22) | 438 (48.3%) | 9,556 (32.7%) | 0.324 | 431 (47.9%) | 414 (46.1%) | 0.038 |
| Occlusion and stenosis of bilateral carotid arteries (I65.23) | 605 (66.8%) | 12,934 (44.2%) | 0.466 | 598 (66.5%) | 620 (69.0%) | 0.052 |
| Occlusion and stenosis of unspecified carotid artery (I65.29) | 492 (54.3%) | 12,355 (42.2%) | 0.243 | 486 (54.1%) | 475 (52.8%) | 0.025 |
| Cerebral infarction (I63) | 437 (48.2%) | 11,539 (39.4%) | 0.178 | 435 (48.4%) | 441 (49.1%) | 0.013 |
| Personal history of TIA and cerebral infarction without residual deficits (Z86.73) | 290 (32.0%) | 5,777 (19.7%) | 0.283 | 288 (32.0%) | 297 (33.0%) | 0.021 |
| Chronic kidney disease (N18) | 323 (35.7%) | 5,712 (19.5%) | 0.367 | 320 (35.6%) | 311 (34.6%) | 0.021 |
| Chronic kidney disease, stage 1 (N18.1) | 13 (1.4%) | 193 (0.7%) | 0.076 | 12 (1.3%) | 10 (1.1%) | 0.020 |
| Chronic kidney disease, stage 2 (mild) (N18.2) | 72 (7.9%) | 922 (3.2%) | 0.211 | 70 (7.8%) | 66 (7.3%) | 0.017 |
| Chronic kidney disease, stage 3 (moderate) (N18.3) | 235 (25.9%) | 3,530 (12.1%) | 0.359 | 233 (25.9%) | 228 (25.4%) | 0.013 |
| Chronic kidney disease, stage 4 (severe) (N18.4) | 42 (4.6%) | 743 (2.5%) | 0.113 | 42 (4.7%) | 43 (4.8%) | 0.005 |
| Chronic kidney disease, stage 5 (N18.5) | 12 (1.3%) | 201 (0.7%) | 0.064 | 12 (1.3%) | 12 (1.3%) | 0.001 |
| Chronic kidney disease, unspecified (N18.9) | 203 (22.4%) | 3,214 (11.0%) | 0.310 | 202 (22.5%) | 205 (22.8%) | 0.008 |
| End stage renal disease (N18.6) | 26 (2.9%) | 503 (1.7%) | 0.077 | 26 (2.9%) | 27 (3.0%) | 0.007 |
| Atrial fibrillation and flutter (I48) | 197 (21.7%) | 4,711 (16.1%) | 0.144 | 194 (21.6%) | 199 (22.1%) | 0.013 |
| <strong>Medications</strong> |  |  |  |  |  |  |
| Aspirin | 820 (90.5%) | 20,764 (71.0%) | 0.511 | 813 (90.4%) | 815 (90.7%) | 0.008 |
| Clopidogrel | 813 (89.7%) | 20,134 (68.8%) | 0.534 | 806 (89.7%) | 825 (91.8%) | 0.073 |
| Ticagrelor | 115 (12.7%) | 1,405 (4.8%) | 0.282 | 112 (12.5%) | 112 (12.5%) | 0.001 |
| Prasugrel | 34 (3.8%) | 457 (1.6%) | 0.137 | 33 (3.7%) | 34 (3.8%) | 0.006 |
| Lisinopril | 458 (50.6%) | 8,440 (28.8%) | 0.455 | 453 (50.4%) | 447 (49.7%) | 0.013 |
| Losartan | 327 (36.1%) | 5,038 (17.2%) | 0.437 | 323 (35.9%) | 335 (37.3%) | 0.028 |
| Atorvastatin | 714 (78.8%) | 15,933 (54.5%) | 0.534 | 707 (78.6%) | 723 (80.4%) | 0.044 |
| Rosuvastatin | 329 (36.3%) | 4,701 (16.1%) | 0.473 | 323 (35.9%) | 294 (32.7%) | 0.068 |
| Simvastatin | 160 (17.7%) | 3,679 (12.6%) | 0.142 | 159 (17.7%) | 160 (17.8%) | 0.003 |
| Pravastatin | 156 (17.2%) | 2,916 (10.0%) | 0.213 | 152 (16.9%) | 139 (15.5%) | 0.039 |
| Fluvastatin | 10 (1.1%) | 42 (0.1%) | 0.122 | 10 (1.1%) | 10 (1.1%) | 0.001 |
| Lovastatin | 21 (2.3%) | 473 (1.6%) | 0.051 | 20 (2.2%) | 21 (2.3%) | 0.007 |
| Pitavastatin | 16 (1.8%) | 164 (0.6%) | 0.113 | 16 (1.4%) | 13 (1.4%) | 0.001 |
| Alirocumab | 16 (1.8%) | 140 (0.5%) | 0.122 | 16 (1.8%) | 13 (1.4%) | 0.026 |
| Evolocumab | 51 (5.6%) | 367 (1.3%) | 0.242 | 45 (5.0%) | 45 (5.0%) | 0.001 |
| Evinacumab | 0 (0%) | 0 (0%) | -- | 0 (0%) | 0 (0%) | -- |
| Ezetimibe | 192 (21.2%) | 2,209 (7.6%) | 0.396 | 187 (20.8%) | 182 (20.2%) | 0.014 |
| <strong>Laboratory Values</strong> |  |  |  |  |  |  |
| Body mass index, kg/m² | 32.5 ± 6.5 | 28.2 ± 5.9 | 0.700 | 32.5 ± 6.5 | 30.2 ± 6.1 | 0.366* |
| eGFR, mL/min/1.73 m² | 69.2 ± 27.2 | 72.2 ± 27.3 | 0.112 | 69.1 ± 27.0 | 68.7 ± 27.2 | 0.012 |
| Hemoglobin A1c, % | 7.5 ± 1.8 | 6.4 ± 1.4 | 0.720 | 7.5 ± 1.8 | 7.0 ± 1.6 | 0.309* |

Values are presented as n (%) for categorical variables and mean ± SD for continuous variables.

Propensity score matching was performed using 1:1 nearest-neighbor matching with a greedy algorithm and caliper of 0.1 pooled standard deviations. Covariates were measured prior to or at the index procedure date.

Standardized mean differences ≤0.10 indicate adequate balance between groups. After matching, all covariates achieved adequate balance (SMD ≤0.10) except for BMI and HbA1c (*), which remained imbalanced (SMD &gt;0.10), with the GLP-1 RA group having higher values for both parameters.*

Abbreviations: BMI = body mass index; eGFR = estimated glomerular filtration rate; GLP-1 RA = glucagon-like peptide-1 receptor agonist; SD = standard deviation; SMD = standardized mean difference; TIA = transient ischemic attack.
